# Open Access in the Age of AI: The Journal of Appalachian Health and Hurricane Helene Recovery

**DOI:** 10.13023/jah.0603.02

**Published:** 2024-10-01

**Authors:** Noah Wren, Bradley Firchow

**Affiliations:** East Tennessee State University

**Keywords:** Appalachia, public health, Hurricane Helene, recovery

## Abstract

The recent destruction brought upon the Appalachian region by Hurricane Helene has emphasized the importance of the *Journal of Appalachian Health*. As an open-access peer-reviewed source of information, the *Journal* plays a critical role in not only facilitating public health research about Hurricane Helene, but also combating misinformation regarding the event. In the days following Hurricane Helene, misinformation about the federal government’s response, along AI generated images, have left many in the region confused and misled about what had happened. Going forward, the *Journal* will play an necessary part in making sure accurate information is shared to understand how we can prepare for future natural disasters, combat misinformation regarding response efforts, and facilitate long-term healing across Appalachia.

The devastation wrought by Hurricane Helene across Appalachia demands a robust, research-driven response to address both immediate needs and long-term recovery. As with previous disasters like Hurricanes Katrina and Sandy, the public health challenges arising from Helene will require careful examination, innovative solutions, and open communication to ensure community well-being and resilience. This editorial argues for the crucial role of the *Journal of Appalachian Health* (*JAH*) in this process, emphasizing the importance of open-access, peer-reviewed research and the need to combat misinformation actively.

The proliferation of misinformation surrounding Hurricane Helene underscores the urgency of providing reliable, evidence-based information to the public. Conspiracy theories regarding land confiscation, fabricated stories about FEMA’s activities, and AI-generated images misrepresenting the extent of damage in specific locales have caused confusion and hindered effective recovery efforts.^2^,^3^,^4^ The ease with which AI tools can create misleading content further complicates the situation, as evidenced by the spread of fabricated articles shared as factual news, regardless of the intent of the person sharing the articles.^3^

Open access journals like *JAH* play a critical role in combating misinformation by providing a platform for rigorously vetted research on health in Appalachia to be disseminated widely and freely. This accessibility ensures that policymakers, practitioners, and the public alike can access accurate information to inform decision-making and guide recovery efforts.^2^ By removing financial barriers, open access fosters greater transparency and collaboration in the scientific community, leading to accelerated research progress and improved public health outcomes.

Previous hurricanes that have hit the United States have left lasting impacts on environmental and public health.^5^,^6^ Research examining these impacts is essential for understanding the long-term consequences of such events and developing effective mitigation strategies.^7^ The *Journal of Appalachian Health*, with its regional focus, is uniquely positioned to become the home for research investigating the environmental and public health challenges emerging from Hurricane Helene.

Potential areas of research include:

○ Evaluating the effectiveness of public health communication strategies employed during the hurricane, analyzing what worked well and what did not. This analysis should examine the role of social media, traditional media outlets, and community-based communication networks.^1^
○ Examining the spread and impact of misinformation on disaster response efforts, exploring strategies to counter false narratives effectively.^7^
○ Investigating the mental health consequences of the hurricane, including the prevalence of PTSD, anxiety, and depression among affected communities pre- and post-disaster.
○ Assessing the impact of the disaster on access to healthcare, including the availability of essential medications, continuity of care for those with chronic illnesses, and the mental health needs of first responders.
○ Determining the impact of the hurricane on long-term excess mortality and morbidity.^8^
○ Lessons learned from responding to and recovering from the hurricane.
○ Personal accounts and ruminations on lived experience during and after the hurricane, particularly pertaining to how it has influenced healthcare providers, patients, first responders, community organizers, and people engaging health systems.
○ Analyzing the long-term environmental health impacts of the hurricane, including water contamination, exposure to hazardous materials, and the potential for increased vector-borne diseases.^7^

Combating misinformation requires a multifaceted approach involving researchers, practitioners, policymakers, and the public. *JAH* can play a critical role in facilitating this effort by:

○ Publishing research that exposes the tactics and sources of misinformation, providing insights into the mechanisms by which false narratives gain traction.^2^
○ Disseminating evidence-based information to the public through accessible channels, utilizing social media, community partnerships, and collaborations with local media outlets.^1^,^2^
○ Providing guidance to public health professionals and organizations on how to identify and address misinformation, equipping them with the tools to respond effectively to false narratives.^2^,^9^,^10^

Hurricane Helene has presented Appalachia with a profound challenge, but it has also created an opportunity to advance our understanding of disaster preparedness, response, and recovery. Helene will not be the last major disaster that faces the region as climate change continues to progress past critical tipping points. The *Journal of Appalachian Health* is poised to play a vital role in this process by serving as a platform for open access research that addresses the multifaceted public health issues arising from the disaster. By embracing open science principles, fostering collaboration, and actively combating misinformation, *JAH* can contribute significantly to building a healthier and more resilient Appalachia in the aftermath of Hurricane Helene.
